# Generation of Anti-Murine ADAMTS13 Antibodies and Their Application in a Mouse Model for Acquired Thrombotic Thrombocytopenic Purpura

**DOI:** 10.1371/journal.pone.0160388

**Published:** 2016-08-01

**Authors:** Louis Deforche, Claudia Tersteeg, Elien Roose, Aline Vandenbulcke, Nele Vandeputte, Inge Pareyn, Elien De Cock, Hanspeter Rottensteiner, Hans Deckmyn, Simon F. De Meyer, Karen Vanhoorelbeke

**Affiliations:** 1 Laboratory for Thrombosis Research, IRF Life Sciences, KU Leuven Campus Kulak Kortrijk, Kortrijk, Belgium; 2 Baxalta Innovations GmbH, Vienna, Austria; Nagoya University, JAPAN

## Abstract

Thrombotic thrombocytopenic purpura (TTP) is a life-threatening thrombotic microangiopathy linked to a deficiency in the metalloprotease ADAMTS13. In the current study, a novel mouse model for acquired TTP was generated to facilitate development and validation of new therapies for this disease. Therefore, a large panel (n = 19) of novel anti-mouse ADAMTS13 (mADAMTS13) monoclonal antibodies (mAbs) of mouse origin was generated. Inhibitory anti-mADAMTS13 mAbs were identified using the FRETS-VWF73 assay. Four mAbs strongly inhibited mADAMTS13 activity *in vitro* (∼68–90% inhibition). Injecting a combination of 2 inhibitory mAbs (13B4 and 14H7, 1.25 mg/kg each) in *Adamts13*^*+/+*^ mice resulted in full inhibition of plasma ADAMTS13 activity (96 ± 4% inhibition, day 1 post injection), leading to the appearance of ultra-large von Willebrand factor (UL-VWF) multimers. Interestingly, the inhibitory anti-mADAMTS13 mAbs 13B4 and 14H7 were ideally suited to induce long-term ADAMTS13 deficiency in *Adamts13*^*+/+*^ mice. A single bolus injection resulted in full *ex vivo* inhibition for more than 7 days. As expected, the mice with the acquired ADAMTS13 deficiency did not spontaneously develop TTP, despite the accumulation of UL-VWF multimers. In line with the *Adamts13*^-/-^ mice, TTP-like symptoms could only be induced when an additional trigger (rVWF) was administered. On the other hand, the availability of our panel of anti-mADAMTS13 mAbs allowed us to further develop a sensitive ELISA to detect ADAMTS13 in mouse plasma. In conclusion, a novel acquired TTP mouse model was generated through the development of inhibitory anti-mADAMTS13 mAbs. Consequently, this model provides new opportunities for the development and validation of novel treatments for patients with TTP. In addition, these newly developed inhibitory anti-mADAMTS13 mAbs are of great value to specifically study the role of ADAMTS13 in mouse models of thrombo-inflammatory disease.

## Introduction

Thrombotic thrombocytopenic purpura (TTP) is a microangiopathic disorder clinically diagnosed by the occurrence of thrombocytopenia and microangiopathic hemolytic anemia [1,2]. The pathophysiology of TTP is linked with a deficiency in the von Willebrand factor (VWF) cleaving protease ADAMTS13 (A Disintegrin And Metalloprotease with ThromboSpondin type-1 repeats, member 13) [3–5]. Under normal physiological conditions, ADAMTS13 cleaves ultra-large von Willebrand factor (UL-VWF) multimers (which are highly reactive in binding platelets) into smaller, quiescent multimers, thereby preventing spontaneous VWF-rich thrombus formation [6–8]. TTP patients suffer from organ ischemia, induced by the presence of these VWF-rich microthrombi in the capillaries and arterioles of mainly the brain, heart and kidneys [7,9]. The majority of TTP patients (∼95%) suffer from acquired TTP caused by the presence of inhibitory and/or clearance-inducing anti-ADAMTS13 autoantibodies (autoAbs) [10,11]. Fewer (<5%) TTP patients have congenital TTP or Upshaw-Schulman Syndrome due to (a) mutation(s) in their *ADAMTS13* gene [12]. Acute disease onset is often induced by a secondary trigger like pregnancy, infection and surgery [13–16].

Suitable TTP animal models have been generated to study the pathophysiology of TTP and to evaluate new therapies for this disease [17]. Animal models for congenital TTP have been developed in mice [18,19] while animal models for acquired TTP have been generated in baboons [20], rats [21] and mice [22]. Importantly, rodents with a congenital or acquired deficiency in ADAMTS13 do not spontaneously develop TTP. Only when rats or mice with a congenital or acquired ADAMTS13 deficiency are triggered with Shiga toxin [18,22] or recombinant VWF (rVWF) [19,20,22], TTP-like symptoms occur. In contrast, baboons with an acquired deficiency in ADAMTS13 spontaneously develop the early symptoms of TTP without the need for an additional trigger [20]. The different animal models for TTP have been exceedingly important in investigating and validating new treatment therapies and in bringing new drugs one-step closer to the clinic. The mouse model for congenital TTP [19] and the rat model for acquired TTP [21] were valuable in demonstrating the efficacy of recombinant ADAMTS13 as a novel therapy for both forms of the disease. The acquired TTP model in baboons proved extremely useful in testing and validating the use of inhibitory anti-VWF Abs as a drug to treat TTP [23,24]. Both recombinant ADAMTS13 and the inhibitory anti-VWF nanobody caplacizumab are currently tested in clinical trials [25].

In the current study we wanted to add a mouse model for acquired TTP to our portfolio of baboon [20] and rat [21] models of acquired TTP. The availability of a small animal model in mice (and rats) is valuable, given the technical (amount of drugs needed, handling, housing and breeding of the animals) and ethical issues related to the use of our large baboon animal model. We generated a large panel (n = 19) of anti-mADAMTS13 mAbs to obtain inhibitory anti-mADAMTS13 mAbs. Injecting the inhibitory mAbs in *Adamts13*^*+/+*^ mice and triggering these mice with rVWF induced TTP-like symptoms. We took the advantage of the availability of our unique anti-mADAMTS13 mAbs to additionally generate a sensitive ELISA to detect plasma mADAMTS13 antigen.

## Materials and Methods

### Mice

ADAMTS13-deficient (*Adamts13*^-/-^) and wild type (WT, *Adamts13*^+/+^) mice were bred from *Adamts13*^*B/CN2-/+*^ animals [18,26]. All animal experiments were performed according to protocols approved by the Institutional Animal Care and Use Committee of the KU Leuven (Belgium). This study was approved under project number P184/2014. Mice were anesthetized using isoflurane/O2. Blood was collected on trisodium citrate (7/1 (vol/vol) of blood/3.8% sodium citrate) and EDTA (15/1 (vol/vol) of blood/0.5 M EDTA) via retro-orbital venipuncture. Total blood cell counts were determined in EDTA anticoagulated blood using the Hemavet 950 (Drew Scientific, Dallas, USA). Plasma was obtained from blood collected on sodium citrate by centrifugation at 2500xg for 6 min and stored at -80°C.

### Construction, expression and purification of mADAMTS13 variants

**Fig 1:**
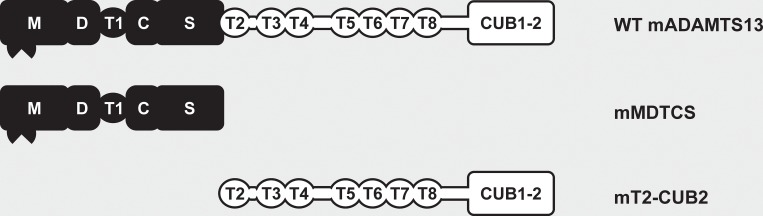
Schematic representation of the mADAMTS13 variants used in this study. The proximal (MDTCS) domains are represented in black and include a metalloprotease (M), disintegrin-like (D), thrombospondin type-1 repeat (T1), cysteine-rich (C) and spacer (S) domain. Distal (T2-CUB2) domains are represented in white and consist of seven thrombospondin type-1 repeats (T2 up to T8) and two CUB (Complement component C1r/C1s, Urinary epidermal growth factor (Uegf) and Bone morphogenic protein-1) domains.

WT mADAMTS13 and its MDTCS variant (mMDTCS) (Fig 1) were described previously [26]. The T2-CUB2 variant (mT2-CUB2) (Fig 1) was constructed by amplifying the cDNA encoding the sequence of mT2-CUB2 from the mADAMTS13-pcDNA6.1 plasmid using Platinum® Pfx DNA polymerase (Invitrogen, Carlsbad, CA, USA). The PCR fragment was cloned in the pSecTag/FRT/V5-His-TOPO® expression vector and the sequence was verified (GATC Biotech AG, Konstanz, Germany). Stable CHO Flp-In cells (Invitrogen) expressing mT2-CUB2 were generated and selected using Hygromycin (Invivogen, San Diego, CA, USA). WT mADAMTS13 and its variant mMDTCS were purified from conditioned medium using a Heparin-Sepharose column (GE Healthcare, Waukesha, WI, USA) and dialysed against HEPES (N-2-hydroxyethylpiperazine-N'-2-ethanesulfonic acid)-buffered saline (HBS) solution (50 mM HEPES, 5 mM CaCl_2_, 1 μM ZnCl_2_ and 150 mM NaCl, pH 7.4), as previously described [27]. The mT2-CUB2 variant was adsorbed on a Ni^2+^ Sepharose Fast Flow column (GE Healthcare). After washing (20 mM NaH_2_PO_4_, 500 mM NaCl, 30 mM imidazole, pH 7.4), bound mT2-CUB2 was eluted with 20 mM NaH_2_PO_4_, 500 mM NaCl, pH 7.4, containing 500 mM imidazole. Fractions were pooled, concentrated and dialysed against HBS.

### Generation of anti-mADAMTS13 mouse mAbs

The anti-mADAMTS13 mouse mAbs were generated by immunizing *Adamts13*^-/-^ mice with recombinant mADAMTS13 in either filter-sterile PBS, complete or incomplete Freund’s adjuvant (Sigma-Aldrich, St Louis, MO, USA), depending on the time point in the immunization procedure. At day 59 (24 h after the last booster injection), the spleen of the immunized mice was isolated and spleen cells were fused with Sp2/0 myeloma cells according to the method of Köhler and Milstein [28] and as previously described [20,29,30]. Anti-mADAMTS13 Ab-producing hybridoma cell lines were identified 14 days after fusion, using ELISA (see below). Positive hybridomas were subcloned to obtain monoclonal cell lines. Hybridomas were diluted on a 96-well microtiter culture plate (Greiner, Frickenhausen, Germany) at 1 cell/well. Two weeks later, positive monoclonal hybridomas were identified. Hybridomas were grown in conditioned medium and anti-mADAMTS13 mAbs were purified using a Protein G Sepharose 4 Fast Flow column (GE Healthcare). The concentration of the purified mAbs was determined by measuring absorbance at 280 nm. Purity was evaluated by SDS-PAGE with subsequent Instant Blue Protein Gel staining (Westburg, Leusden, The Netherlands). Different batches of antibody were used, where 13B4 had a concentration ranging from 0.85 to 1.33 mg/mL and 14H7 had a concentration ranging from 0.92 to 1.20 mg/mL.

### Identification of anti-mADAMTS13 mAb-producing hybridoma cell lines

Anti-mADAMTS13 mAb-producing hybridomas were identified using ELISA. In brief, purified recombinant mADAMTS13 was coated overnight at 1 U/mL (1 U/mL corresponds to the concentration of mADAMTS13 in normal murine plasma [NMP] of *Adamts13*^*+/+*^ mice) on a 96-well microtiter plate. After blocking (3% milk in PBS), hybridoma supernatant was added. Bound mAbs were detected after 1 h with horseradish peroxidase (HRP)-labeled goat anti-mouse immunoglobulin G (IgG), Fc-specific (GAM-HRP, Sigma-Aldrich) (1/10,000 in PBS, 0.3% milk). Colorimetric development was done using o-phenylenediamine dihydrochloride (OPD) and H_2_O_2_, and was stopped using 4 M sulfuric acid, after which the absorbance at 490 nm was determined.

### Epitope mapping of the anti-mADAMTS13 mAbs

The epitope of all anti-mADAMTS13 mAbs was mapped against recombinant mMDTCS and mT2-CUB2. Briefly, a 96-well microtiter plate was coated overnight with the respective anti-mADAMTS13 mAb (6 μg/mL). After blocking, 15 nM of mMDTCS or mT2-CUB2 was added for 1 h at 37°C. Binding was detected using the in-house developed polyclonal rabbit anti-mADAMTS13 Ab (5 μg/mL) [31] and goat anti-rabbit IgG labelled with HRP (GAR-HRP, Jackson ImmunoResearch, West Grove, PA, USA) (1/20,000 in PBS 0.3% milk). Colorimetric development was performed as described above.

### Development of a sensitive mADAMTS13 antigen detection assay

Detection of plasma and recombinant mADAMTS13 using ELISA was previously described [31] and further optimized by changing the capturing mAb. Therefore, the anti-mADAMTS13 mAbs were coated separately overnight on a 96-well microtiter plate (5 μg/mL). After blocking, a serial dilution of NMP of *Adamts13*^*+/+*^ mice (initial concentration of 0.1 U/mL) was added for 1 h at 37°C. Detection of binding was done using the in-house developed polyclonal rabbit anti-mADAMTS13 Ab (5 μg/mL) [31] and GAR-HRP. Colorimetric development was performed as described above.

### Activity of plasma mADAMTS13

Both the *in vitro* and *ex vivo* effect of the anti-mADAMTS13 mAbs on the proteolytic activity of plasma mADAMTS13 was tested in the FRETS-VWF73 assay as previously described [31].

The *in vitro* effect was tested by addition of the mAbs (10 μg/mL) and 15% NMP to 2 μM of the FRETS-VWF73 substrate solution in HBS (pH 7.4) with 1 mg/mL BSA (bovine serum albumin, Sigma-Aldrich). In addition, the activities of 2.5%, 5%, 10% and 15% NMP in the absence of mAbs were tested to set-up a calibration curve. Generation of cleavage product was analysed by measuring fluorescence intensities with the FLUOstar OPTIMA (BMG Labtech GmbH, Offenburg, Germany) every 150 s for 75 min using excitation at 355 nm and emission at 460 nm at 37°C. The proteolytic activity of plasma mADAMTS13 was determined by calculating the slope of the cleavage reaction and comparing it to the calibration curve.

The *ex vivo* effect on the proteolytic activity of plasma mADAMTS13 of the individual mAbs 13B4, 14H7 and 20A10 (all directed against mMDTCS), and the combined effect of mAbs 13B4 and 14H7 was initially tested through retro-orbital injection (2.50 mg/kg for the individual mAbs and 1.25 mg/kg for 13B4 and 14H7) in *Adamts13*^+/+^ mice (n = 4 for each condition). Blood was taken 7 days before injection and 1, 3, 5, 7 and 14 days post injection. The proteolytic activity of plasma mADAMTS13 was determined using the FRETS-VWF73 assay, as described above.

### Ab plasma levels in the treated *Adamts13*^+/+^ mice

The plasma concentration of the mAbs in the *Adamts13*^*+/+*^ mice treated with the respective anti-mADAMTS13 mAb(s) was determined using the above described ELISA for the identification of the anti-mADAMTS13 mAb-producing hybridoma cell lines, with some minor modifications. Briefly, purified recombinant mADAMTS13 was coated overnight at 1 U/mL on a 96-well microtiter plate. As a calibration curve, a constant amount of NMP (1/10) with a ½ dilution of the respective mAb (13B4 and/or 14H7 or 20A10, initial concentration of 2.5 μg/mL) was added after blocking. Plasma samples of the injected mice (7 days before injection and 1, 3, 5, 7 and 14 days post injection) were added in a ½ dilution (1/10 dilution in the first well). Bound mAbs were detected after 1 h with GAM-HRP. Colorimetric development was performed as described above. The half-life of the mAbs was determined by fitting the calculated plasma concentrations in function of time, using a logarithmic regression curve.

### VWF multimer analysis

The effect of injection of mAb 20A10 or the combination of mAbs 13B4 and 14H7 on the plasma murine VWF (mVWF) multimer pattern of the treated *Adamts13*^*+/+*^ mice (described above) was analysed as described [32,33]. Plasma samples of the injected mice (7 days before injection and 1 and 3 days post injection) were loaded on 1.2% IEF agarose gels. After separation of plasma mVWF, gels were fixed on Gelbond (Westburg). Next, plasma mVWF was detected using the anti-VWF-Ig labelled with alkaline phosphatase (AP) (Dako, Heverlee, Belgium) and an AP conjugate substrate kit (Biorad, Hercules, CA, USA). Multimer densitometry was performed using the ImageJ 1.48v software.

### Mouse model for acquired TTP

Acquired deficiency of ADAMTS13 in *Adamts13*^+/+^ mice was obtained by injecting both inhibitory anti-mADAMTS13 mAbs 13B4 and 14H7 (both 1.25 mg/kg) in 6 male and 6 female mice (age of 8 weeks) at day -1. The non-functional anti-mMDTCS mAb 20A10 was injected (2.50 mg/kg) in 5 male and 5 female *Adamts13*^+/+^ mice (age of 8 weeks) as negative control. After 24 h (day 0), all mice were injected with recombinant human VWF (rVWF; 500 U/kg) via the tail vein to trigger TTP. Blood and plasma were taken 7 days before injection and 1, 2, 4 and 14 days post injection. Subsequently, platelet counts were measured (Hemavet), lactate dehydrogenase (LDH) activity (Gentaur [Biovision], SanJose, CA, USA) was determined and the multimeric pattern was analysed as described above.

### Statistical analysis

Slopes of the data of the FRETS-VWF73 assay were fitted through linear regression. Means were compared with the reference, using the Student’s *t*-test (parametric, unpaired, 2-tailed). Probabilities of *P* < 0.05 (*), *P* < 0.01 (**), *P* < 0.001 (***) and P < 0.0001 (****) were taken as significant for rejection of the null hypothesis. All statistical applications were performed using the GraphPad Prism v5.03 software (GraphPad Software, Inc., San Diego, CA, USA).

## Results

### Development of anti-mADAMTS13 mAbs

**Fig 2:**
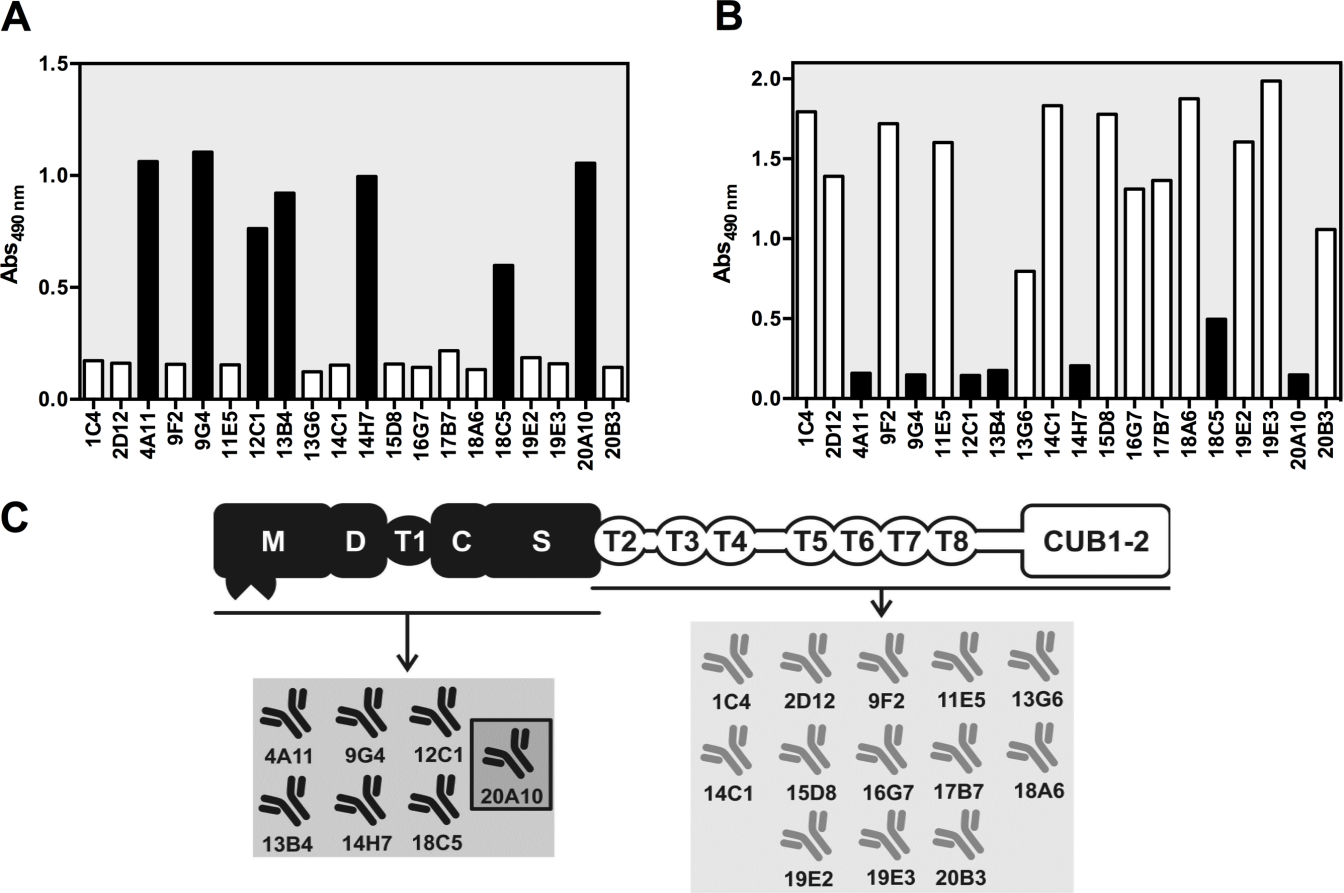
Epitope mapping and epitope overview of the developed anti-mADAMTS13 mAbs. The epitope of each anti-mADAMTS13 mAb was mapped against both mMDTCS (A) and mT2-CUB2 (B). Individual anti-mADAMTS13 mAbs were coated, recombinant mMDTCS (A) or mT2-CUB2 (B) were added and binding of the respective mADAMTS13 variant was detected using the polyclonal anti-mADAMTS13 rabbit IgG and GAR-HRP. Black and white bars represent respectively anti-mMDTCS and anti-mT2-CUB2 mAbs. The previously reported mAb 20A10 [31] was used as a positive (A) and negative (B) control. (C) Epitope overview of the developed anti-mADAMTS13 mAbs. The previously developed mAb 20A10 [31] is marked by a dark frame.

We aimed at generating a large panel of anti-mADAMTS13 mAbs to obtain inhibitory mAbs in order to develop an acquired ADAMTS13 deficiency in mice. In addition, novel (non-inhibitory) anti-mADAMTS13 mAbs could be essential new tools to detect mADAMTS13 antigen in murine plasma. Hence, *Adamts13*^-/-^ mice were immunized with recombinant mADAMTS13, B-cells were fused with Sp2/0 myeloma cells and hybridoma cells expressing anti-mADAMTS13 mAbs were selected. This led to the generation of 19 anti-mADAMTS13 mAbs. To identify anti-amino(N)-terminal (MDTCS) or anti-carboxyl(C)-terminal (T2-CUB2) mAbs (Fig 1), anti-ADAMTS13 mAbs were mapped against recombinant mMDTCS and mT2-CUB2. Six mAbs had an epitope in the MDTCS part (Fig 2A) and 13 had an epitope in the T2-CUB2 part (Fig 2B). A schematic overview of the epitopes of all anti-mADAMTS13 mAbs is given in Fig 2C.

### Generation and validation of a sensitive mADAMTS13 antigen assay

**Fig 3:**
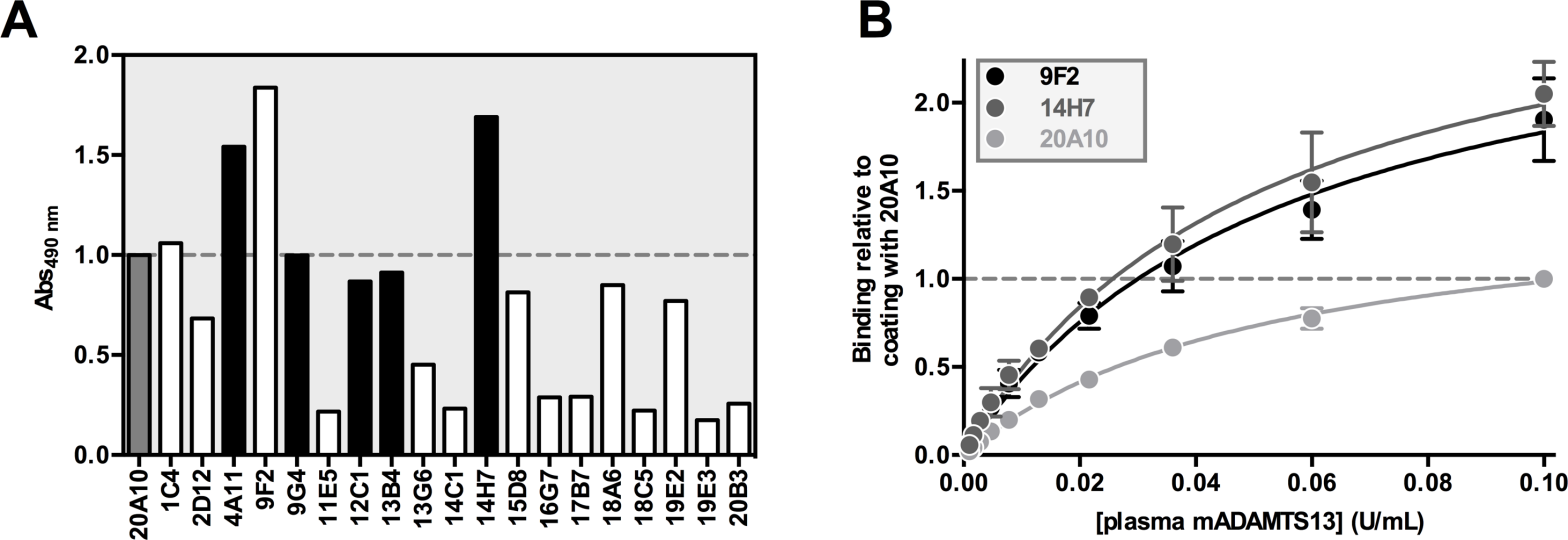
Development of a sensitive mADAMTS13 detection assay. (A) Binding of plasma mADAMTS13 to the anti-mADAMTS13 mAbs was tested in ELISA. The respective anti-mMDTCS (black) and anti-mT2-CUB2 (white) mAbs were coated on a 96-well microtiter plate. After blocking, plasma mADAMTS13 was added (0.1 U/mL) and was detected using the polyclonal anti-mADAMTS13 rabbit IgG and GAR-HRP. (B) The same ELISA was performed as in A in triplicate for anti-mT2-CUB2 mAb 9F2, anti-mMDTCS mAb 14H7 and the previously reported anti-mMDTCS mAb 20A10 [31]. Binding was calculated relative to binding of 0.10 U/mL plasma mADAMTS13 to 20A10 (which was set to a value of ‘1.0’). Error bars represent the SD of the three independently performed experiments.

In our first attempt to generate anti-mADAMTS13 mAbs, only a single anti-mADAMTS13 mAb (mAb 20A10) was obtained [31]. With this and an in-house developed polyclonal anti-mADAMTS13 rabbit IgG [31], we succeeded in developing the first ELISA to detect plasma mADAMTS13. The 19 novel anti-mADAMTS13 mAbs created the opportunity to improve the sensitivity of this unique assay. All mAbs, including 20A10, were coated and their capacity to capture plasma mADAMTS13 was evaluated. Anti-mMDTCS mAb 14H7 and anti-mT2-CUB2 mAb 9F2 were found most efficient in capturing plasma mADAMTS13 (Fig 3).

The efficacy of the three anti-mADAMTS13 mAbs (9F2, 14H7 and 20A10) in capturing plasma mADAMTS13 was further evaluated by determination of the repeatability, reproducibility, detection limit (DL) and quantification limit (QL) of the three respective plasma mADAMTS13 detection assays. The repeatability was determined by testing ten dilutions (1/5 to 1/2560) of NMP on six replicates in one run. Coefficients of variation (CV; calculated as standard deviation divided by the mean, multiplied by 100%) ranged from 2 to 9% for 9F2, 3 to 9% for 14H7 and 3 to 7% for 20A10 (Table 1). The reproducibility was calculated by analysing six replicates of ten dilutions (1/5 to 1/2560) of NMP in three runs by two technicians. The CV ranged from 4 to 23% for 9F2, 4 to 16% for 14H7 and 5 to 17% for 20A10 (Table 1). The DL (defined as 3x SD above the mean zero standard) of plasma mADAMTS13 was a dilution of 1/640 (1.56 mU/mL), 1/1280 (0.78 mU/mL) and 1/320 (3.1 mU/mL) for 9F2, 14H7 and 20A10 respectively. The QL (defined as the minimum concentration that can be measured from assay to assay with CV < 20%) was equal to the DL.

**Table 1:** Validation of the mADAMTS13 detection ELISA’s.

	Repeatability (CV, %)			Reproducibility (CV, %)		
Dilution of NMP	9F2	14H7	20A10	9F2	14H7	20A10
1/5	2	3	3	4	5	7
1/10	3	3	3	4	5	6
1/20	4	4	4	5	6	6
1/40	4	4	3	9	7	5
1/80	5	4	7	6	5	17
1/160	4	3	5	5	4	8
1/320	5	5	5	11	14	6
1/640	5	4	5	5	7	6
1/1280	6	9	6	13	16	7
1/2560	9	6	6	23	8	8

Anti-mADAMTS13 mAbs 9F2, 14H7 and 20A10 were coated, different dilutions of NMP were added and bound plasma mADAMTS13 was detected with the polyclonal anti-mADAMTS13 rabbit IgG and GAR-HRP. The CV (%) was calculated for each dilution.

In conclusion, the novel panel of 19 anti-mADAMTS13 mAbs resulted in the development of a more sensitive ADAMTS13 detection ELISA, using either the anti-mMDTCS mAb 14H7 (DL and QL of 0.78 mU/mL) or the anti-mT2-CUB2 mAb 9F2 (DL and QL of 1.56 mU/mL) as a capturing mAb, compared to the use of the anti-mMDTCS mAb 20A10 (DL and QL of 3.1 mU/mL).

### In vitro identification of inhibitory anti-mADAMTS13 mAbs

**Fig 4:**
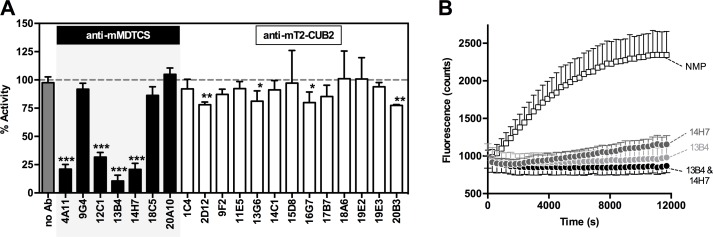
A subset of anti-mMDTCS mAbs inhibit plasma mADAMTS13 activity. The effect of anti-mADAMTS13 mAbs on the proteolytic activity of plasma mADAMTS13 was tested *in vitro* using the FRETS-VWF73 assay. (A) All anti-mADAMTS13 mAbs, both anti-mMDTCS and anti-mT2-CUB2 mAbs, were tested. For each condition, the activity of plasma mADAMTS13 was calculated using a calibration curve of 2.5%, 5%, 10% and 15% NMP. (B) Time profile of the cleavage of the FRETS-VWF73 substrate by plasma mADAMTS13, in the absence or presence of mAbs 13B4 and/or 14H7. Error bars represent the SD of at least three independently performed experiments.

In order to obtain acquired deficiency in *Adamts13*^*+/+*^ mice, inhibitory anti-mADAMTS13 mAbs had to be identified. Hence, all mAbs were tested in the FRETS-VWF73 assay to evaluate their inhibitory effect on the proteolytic activity of plasma mADAMTS13. Four anti-mMDTCS mAbs strongly inhibited mADAMTS13 activity (∼68–90% inhibition) while 4 anti-mT2-CUB2 mAbs showed a weak inhibitory effect (∼20% inhibition) (Fig 4A). Full inhibition of mADAMTS13 activity (100%) was obtained by combining mAbs 13B4 and 14H7 (Fig 4B).

### Ex vivo characterization of inhibitory anti-mADAMTS13 mAbs 13B4 and 14H7

**Fig 5:**
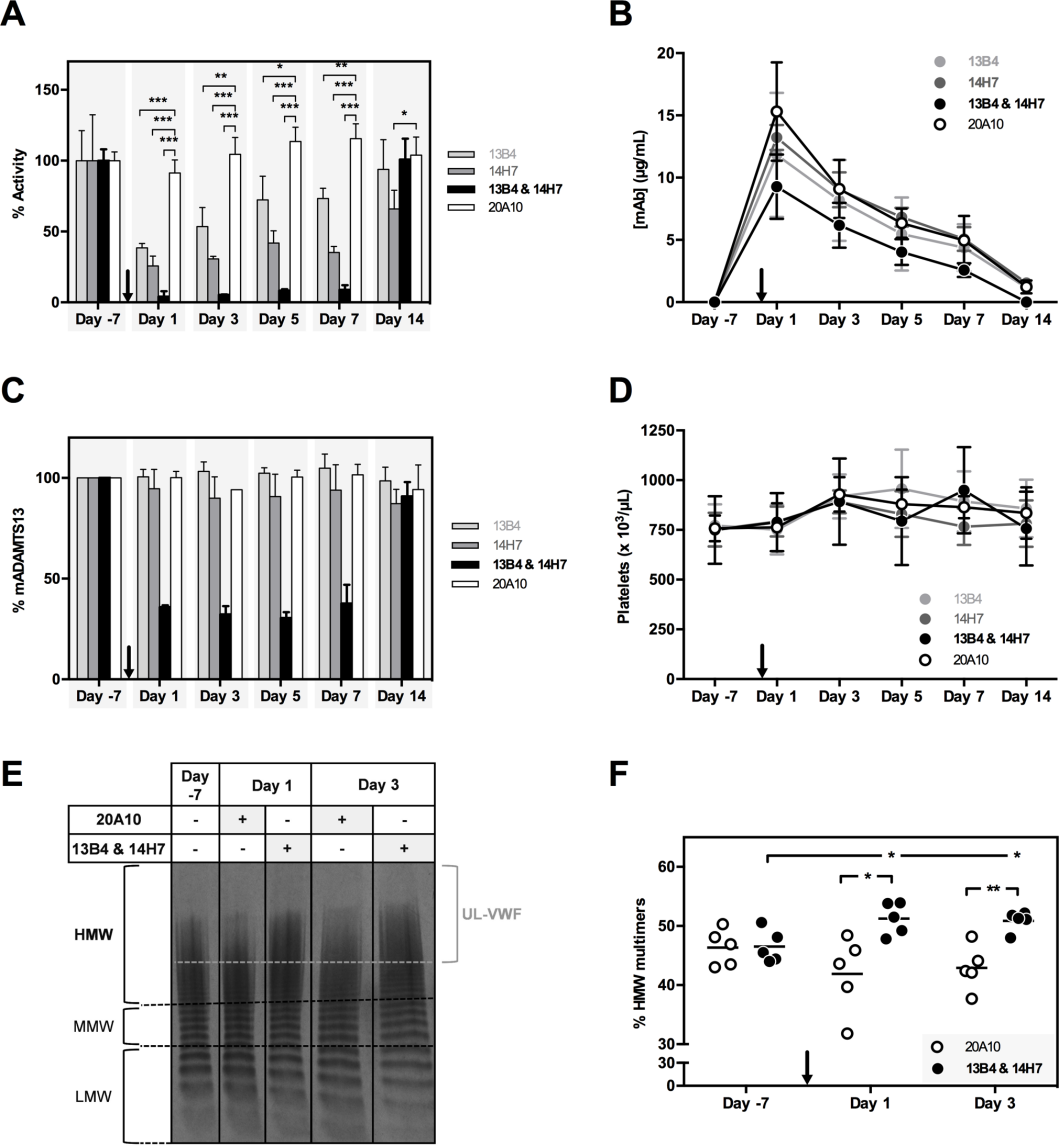
Characterization of the *ex vivo* inhibitory effect of anti-mMDTCS mAbs 13B4 and 14H7. *Adamts13*^*+/+*^ mice (n = 4, per condition) were injected with 2.50 mg/kg of mAb 13B4, 14H7 or 20A10 or with a combination of mAbs 13B4 and 14H7 (1.25 mg/kg each) on day 0 (black arrow). The optimal injection dose of mAb was determined in separate experiments (data not shown). Blood was retrieved 7 days before (‘day -7’) and 1, 3, 5, 7 and 14 days post injection. (A) The influence of the different mAbs on the proteolytic activity of mADAMTS13 was determined using the FRETS-VWF73 assay. Activities were calculated based on the slope of the proteolysis reactions (S1 Fig). (B) Plasma mAb levels (μg/mL) were determined using ELISA. Plates were coated with recombinant mADAMST13, blocked and plasma of the respective mice was added. Bound mAbs were detected using GAM-HRP. (C) The amount of mADAMTS13 (%) in plasma was determined using ELISA. Plasma mADAMTS13 was captured using the anti-mT2-CUB2 mAb 9F2. After blocking, the respective plasma samples were added. Finally, bound mADAMTS13 was detected using the polyclonal anti-mADAMTS13 rabbit IgG and GAR-HRP. (D) Platelet counts were measured of the respective mice samples. Error bars represent the SD (n = 4, per condition). (E) The plasma mVWF multimer pattern was determined for a new cohort of treated mice (n = 5, per condition) 7 days before (‘day -7’) and 1 and 3 days post injection of mAb(s) 20A10 or the combination of mAbs 13B4 and 14H7). Representative multimer patterns are given. Low, middle and high molecular weight (respectively LMW [1–5 bands], MMW [6–10 bands] and HMW [>10 bands]) multimers and UL-VWF multimers (brace) are indicated. (F) The percentage HMW multimers was calculated using the ImageJ 1.48v software.

We next investigated the *ex vivo* effect of inhibitory mAbs 13B4 and 14H7 by injecting a dose of 2.50 mg/kg in *Adamts13*^+/+^ mice. Mice injected with the non-inhibitory mAb 20A10 (2.50 mg/kg) were used as a control. When either anti-mADAMTS13 mAb 13B4 or 14H7 was injected in *Adamts13*^+/+^ mice, only partial inhibition of mADAMTS13 activity was observed (at day 1, 3, 5 and 7 post injection, respectively 62 ± 3%, 47 ± 13%, 28 ± 17% and 27 ± 7% inhibition for 13B4; and 74 ± 7%, 69 ± 2%, 58 ± 9% and 65 ± 4% inhibition for 14H7, Fig 5A and S1 Fig). At 14 days post injection, mADAMTS13 activity was fully recovered in mice injected with 13B4 but not in mice injected with 14H7 (6 ± 21% and 34 ± 13% inhibition respectively, Fig 5A and S1 Fig). Injection of the non-inhibitory mAb 20A10 did not affect mADAMTS13 activity *ex vivo* (Fig 5A and S1 Fig). On the other hand, administration of the combination of 13B4 and 14H7 (1.25 mg/kg each) resulted in full inhibition of mADAMTS13 *ex vivo* for up to 7 days (96 ± 4%, 94.7 ± 0.4%, 92 ± 1% and 91 ± 3% inhibition at day 1, 3, 5 and 7 days post injection respectively, Fig 5A and S1 Fig). At day 14 post injection, mADAMTS13 function was fully restored (101 ± 15% activity, Fig 5A and S1 Fig). The long-lasting inhibition of mADAMTS13 activity coincided with the long half-life of the injected mAbs (respectively 4.7 ± 0.5 days, 4.9 ± 0.7 days, 4.1 ± 0.4 days and 4.3 ± 0.7 days for mAb 13B4, 14H7, the combination of 13B4 and 14H7 and the individual mAb 20A10, Fig 5B). In addition, mADAMTS13 antigen levels did not change upon injection of 13B4, 14H7 or 20A10 (Fig 5C) while a reduction of ∼70% in mADAMTS13 antigen was observed when both mAbs 13B4 and 14H7 were injected, which was normalized 14 days post injection (Fig 5C). These data show that the acquired deficiency of ADAMTS13 induced by injecting both mAbs 13B4 and 14H7 is the combined result of inhibition and clearance of ADAMTS13. As expected in view of what is observed in *Adamts13*^*-/-*^ mice [18,34], full inhibition of mADAMTS13 function in *Adamts13*^+/+^ mice did not result in TTP as platelet counts did not change in these mice (Fig 5D). Nevertheless, analysis of the VWF multimer pattern (Fig 5E and 5F) revealed an increase in high molecular weight (HMW) multimers on day 1 and day 3 post injection of both mAbs 13B4 and 14H7 compared to VWF multimers on day -7 or of mice injected with mAb 20A10, supporting the achievement of an acquired ADAMTS13 deficiency in these mice. As observed for *Adamts13*^-/-^ mice, additional triggers like recombinant hVWF [19] or Shiga toxin [18] are needed to induce TTP in mice with an acquired ADAMTS13 deficiency.

### Acquired TTP mouse model

**Fig 6:**
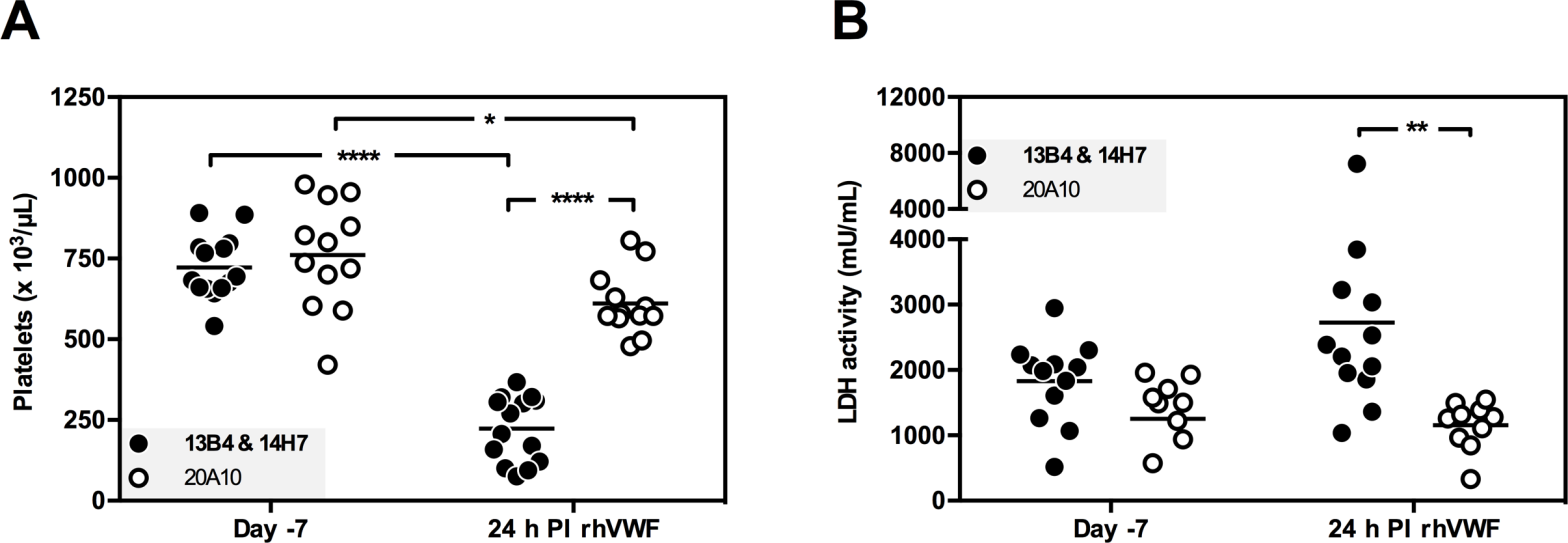
Mouse model for acquired TTP. The mAbs 20A10 (2.50 mg/kg) or 13B4 and 14H7 (1.25 mg/kg each) were retro-orbitally injected in *Adamts13*^*+/+*^ mice at day -1. TTP was induced by intravenous tail injection of rVWF (500 U/kg) at day 0. Blood sampling was performed 7 days before initiating the experiment (‘day -7’) and 24 h post injection of rVWF (‘24 h PI rhVWF’). (A) Platelet counts and (B) LDH activity levels were measured. Each dot represents the value for a single mouse (n ≥ 12).

To develop an acquired TTP mouse model, animals with an acquired deficiency in mADAMTS13 were therefore additionally triggered using rVWF [19]. Twenty-four hours after (‘day 0’) injection of both mAb 13B4 and 14H7 (1.25 mg/kg each) or the non-inhibitory mAb 20A10 (2.50 mg/kg) as a control (n ≥ 12, ‘day -1’), all mice were triggered with 500 U/kg rVWF. Blood and plasma were taken 7 days before (‘day -7’) the experiment and at day 1, 2, 4 and 14. *Adamts13*^+/+^ mice injected with both 13B4 and 14H7 and triggered with rVWF developed severe thrombocytopenia (223 ± 101 x 10^3^ platelets/μL compared to 723 ± 99 x 10^3^ platelets/μL before injection of the trigger, Fig 6A) in contrast to mice injected with the control mAb 20A10 (611 ± 99 x 10^3^ platelets/μL compared to 760 ± 168 x 10^3^ platelets/μL before injection of the trigger, Fig 6A). Thrombocytopenia persisted for 48 h in *Adamts13*^+/+^ mice injected with 13B4 & 14H7 (321 ± 65 x 10^3^ platelets/μL, S2 Fig) and recovered 4 days after the injection with rVWF (S2 Fig). Two weeks after injection of rVWF (day 14), the platelet number was restored to the normal physiological level (S2 Fig). Also lactate dehydrogenase (LDH) activity, a marker for tissue damage present during TTP [35], was increased in mice injected with 13B4 & 14H7 compared to mice treated with 20A10 (Fig 6B).

In conclusion, a murine model for acquired TTP was established by inhibition of mADAMTS13 activity using inhibitory anti-mADAMTS13 mAbs and by subsequently triggering the mice with recombinant hVWF.

## Discussion

In this study, we present a mouse model for acquired TTP by injecting a combination of the two inhibitory mAbs 13B4 and 14H7, selected from our large panel (n = 19) of novel anti-mADAMTS13 mAbs (Figs 1 and 4). A single dose injection of mAbs 13B4 and 14H7 in *Adamts13*^*+/+*^ mice resulted in an acquired deficiency of plasma mADAMTS13 and an increase in HMW VWF multimers (Fig 5). Acquired ADAMTS13 deficiency was due to complete inhibition of the proteolytic activity and partial clearance of mADAMTS13 (Fig 5). Finally, onset of TTP (reflected by thrombocytopenia and elevated LDH activity, Fig 6) was triggered through the tail vein injection of rVWF [19]. A mouse model for acquired TTP where ADAMTS13 deficiency is the result of both inhibition and clearance of the enzyme is in line with the type of acquired ADAMTS13 deficiency observed in humans. Indeed, besides the presence of inhibitory anti-ADAMTS13 antibodies in patients’ plasma, Thomas *et al*. recently suggested that ADAMTS13 clearance might be a major pathological mechanism in acquired TTP [36].

Our mouse model of acquired TTP expands our portfolio of acquired rat and baboon TTP models [20,21]. Investing in different animal models of acquired TTP is essential since novel treatment strategies have to be carefully evaluated in different types of animal models before they can safely enter clinical trials. In addition, each type of animal model has its own advantages and disadvantages [17]. The small rodent models of acquired TTP allow high throughput screening of novel drugs since mice and rats are easy to handle, breed and house and only small amounts of drugs are needed. On the other hand, due to their evolutionary distance from humans and due to the need of additional triggers such as rVWF and Shiga toxin, translation of data obtained in mice and rats to humans is not always evident. The novel mouse model might be better suited to study the treatment efficacy of new drugs than the rat model as TTP symptoms last for 48h in the mouse (Fig 6) compared to 24h in the rat [21]. This shows that the time window to study treatment for acquired TTP is larger in mice compared to rats. The baboon model [20] is less straightforward to use compared to the mouse and rat models because of ethical issues, more complicated handling, breeding and housing and large amount of drugs that are needed. Nevertheless, if novel drugs do not cross-react with mouse or rat ADAMTS13 and/or an animal is needed that is evolutionary closely related to humans, baboons are crucial in drug development. A limitation of all animal models of acquired TTP however, is that injection of the inhibitory anti-ADAMTS13 antibodies does not reflect the continuous production of anti-ADAMTS13 antibodies as in acquired TTP patients.

The long-term inhibitory properties of anti-mADAMTS13 mAbs 13B4 and 14H7 (up to seven days with one bolus injection, Fig 5) create the possibility of inactivating ADAMTS13 in any type of knock out (KO) mouse without the need of crossing these KO mice with *Adamts13*^-/-^ mice, which is highly time- and labour-intensive. The inhibitory mAbs 13B4 and 14H7 will additionally create great potential to determine the role of ADAMTS13 in other diseases besides TTP. Indeed, ADAMTS13 seems to play a role in thromboinflammatory diseases like atherosclerosis [37], myocardial infarction [38–41], ischemic stroke [40,42], preeclampsia [43,44] and cerebral malaria [45].

Finally, our effort to generate mAbs also resulted in the improvement of the sensitivity of the plasma mADAMTS13 detection assay [31]. Capturing plasma mADAMTS13 with the anti-mMDTCS mAb 14H7 or anti-mT2-CUB2 mAb 9F2 increased the sensitivity from 3.1 mU/mL (with mAb 20A10 as coating) to respectively 0.78 mU/mL and 1.56 mU/mL. In addition to the increased sensitivity, both assays are also able to detect plasma mADAMTS13 from mouse strains expressing the short form of mADAMTS13, which lacks the C-terminal mT7-CUB2 domains (data not shown) [46].

In conclusion, we described a novel mouse model for acquired TTP, using the well-characterized inhibitory anti-mADAMTS13 mAbs 13B4 and 14H7. In line with the rat model for acquired TTP, this mouse model creates the opportunity to further investigate novel treatment strategies for acquired TTP before performing large animal studies. Furthermore, these mAbs can also be used to further study the role of ADAMTS13 in other diseases beyond TTP.

## Supporting Information

S1 Fig *Ex vivo* inhibition of plasma mADAMTS13 activity by mAbs 13B4 and 14H7. *Adamts13*^*+/+*^ mice (n = 4, per condition) were injected with 2.50 mg/kg of mAb 13B4, 14H7 or 20A10 or with both mAbs 13B4 and 14H7 (1.25 mg/kg each). Plasma was retrieved 7 days before (‘day -7’) and 1, 3, 5, 7 and 14 days post injection. The influence of the respective mAbs on the proteolytic activity of mADAMTS13 was determined using the FRETS-VWF73 assay. Slopes were calculated using linear regression and used for activity determination (Fig 5A). Error bars represent the SD (n = 4, per condition).
(TIFF)

S2 Fig Time course of platelet counts in the acquired TTP mouse model. Mice injected (‘day -1’) with mAbs 20A10 (white dots) or 13B4 & 14H7 (black dots) were triggered with 500 U/kg rVWF on day 0 (grey arrow, ‘rhVWF’). Platelet counts were measured 6 days before injection (‘day -7’) of the mAbs and after injection of rVWF. Each dot represents the value for a single mouse (n ≥ 8).
(TIFF)
