# Supplementary figures and images for: Generation of Anti-Murine ADAMTS13 Antibodies and Their Application in a Mouse Model for Acquired Thrombotic Thrombocytopenic Purpura
Source: PLoS One. 2016 Aug 1;11(8):e0160388. doi: 10.1371/journal.pone.0160388 (PMC4968830; doi:10.1371/journal.pone.0160388)

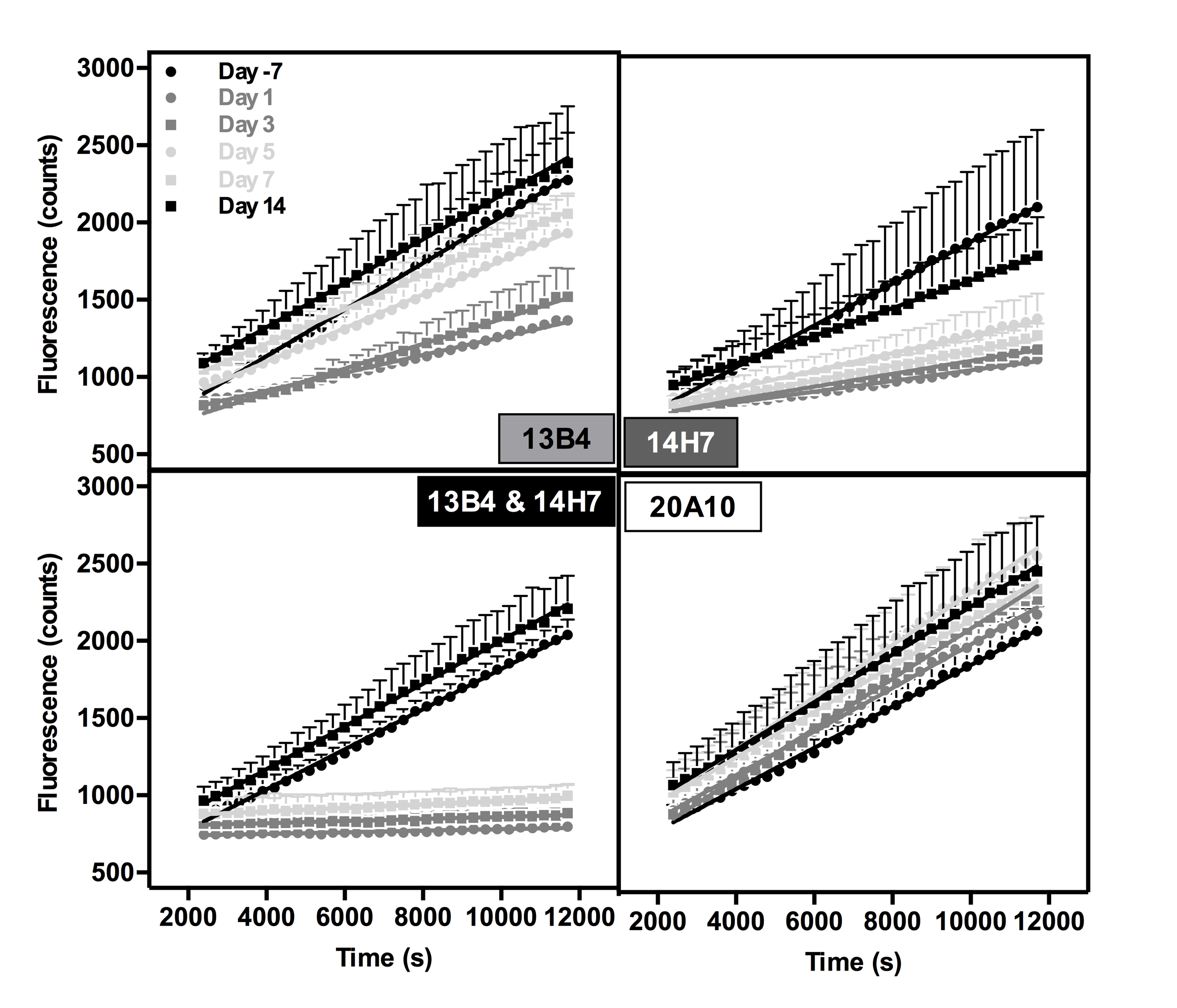

Supplement: S1 Fig — Adamts13+/+ mice (n = 4, per condition) were injected with 2.50 mg/kg of mAb 13B4, 14H7 or 20A10 or with both mAbs 13B4 and 14H7 (1.25 mg/kg each). Plasma was retrieved 7 days before (‘day -7’) and 1, 3, 5, 7 and 14 days post injection. The influence of the respective mAbs on the proteolytic activity of mADAMTS13 was determined using the FRETS-VWF73 assay. Slopes were calculated using linear regression and used for activity determination (Fig 5A). Error bars represent the SD (n = 4, per condition). (TIFF) [file pone.0160388.s001.tiff]

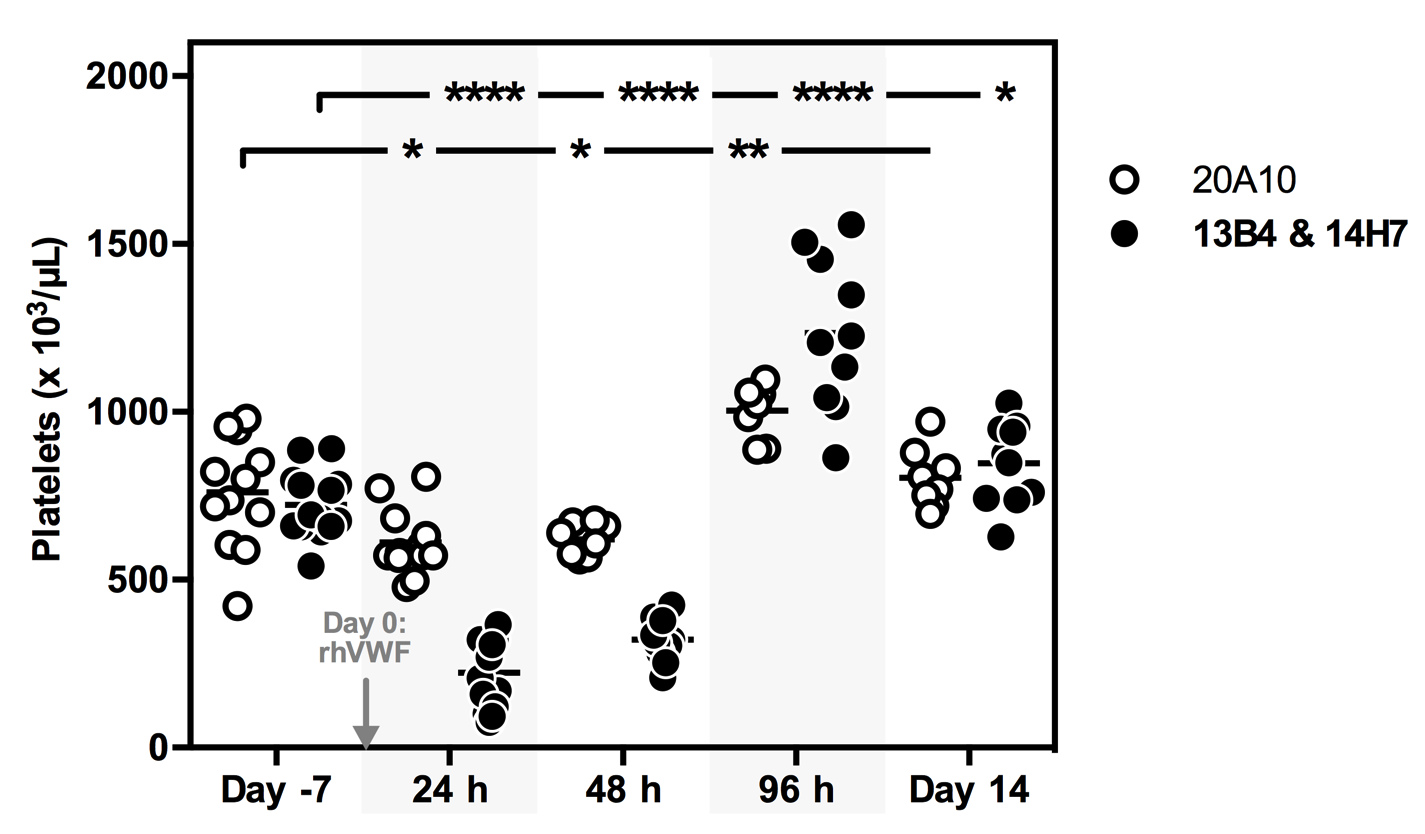

Supplement: S2 Fig — Mice injected (‘day -1’) with mAbs 20A10 (white dots) or 13B4 & 14H7 (black dots) were triggered with 500 U/kg rVWF on day 0 (grey arrow, ‘rhVWF’). Platelet counts were measured 6 days before injection (‘day -7’) of the mAbs and after injection of rVWF. Each dot represents the value for a single mouse (n ≥ 8). (TIFF) [file pone.0160388.s002.tiff]
